# Efficiency of passive activated carbon anaesthetic gas capturing systems during simulated ventilation

**DOI:** 10.1016/j.bja.2024.05.028

**Published:** 2024-07-02

**Authors:** Christin Wenzel, Bernd Flamm, Torsten Loop, Stefan Schumann, Johannes Spaeth

**Affiliations:** Department of Anesthesiology and Critical Care, University of Freiburg Medical Center, Faculty of Medicine, University of Freiburg, Freiburg, Germany

**Keywords:** gas capturing systems, passive flow systems, sevoflurane, sustainability, volatile anaesthetic

## Abstract

**Background:**

Interest in passive flow filter systems to remove sevoflurane from anaesthetic machine exhaust have increased recently to mitigate the environmental impact of volatile anaesthetics. These filter systems consist of chemically activated carbon, with limited evidence on their performance characteristics. We hypothesised that their efficiency depends on filter material.

**Methods:**

Binding capacity was tested for three carbon filter materials (CONTRAfluran®, FlurAbsorb®, and Anaesthetic Agent Filter AAF633). Adsorption efficiency and resistive pressure were determined during simulated ventilation at different stages of filter saturation and fresh gas flow. In addition, sevoflurane concentration in filtered gas was measured at randomly selected anaesthesia workstations.

**Results:**

Sevoflurane concentration in filtered gas exceeded 10 ppm when saturated with 184 ml sevoflurane each for CONTRAfluran and FlurAbsorb and 276 ml for AAF633. During simulated ventilation, sevoflurane concentration >10 ppm passed through CONTRAfluran and AAF633 at fresh gas flow 10 L min^−1^ only at maximum saturation, but through FlurAbsorb at all stages of saturation. The resistance pressure of all filters was negligible during simulated ventilation, but increased up to 5.2 (0.2) cm H_2_O during simulated coughing. At two of seven anaesthesia workstations, sevoflurane concentration in filtered exhaust gas was >10 ppm.

**Conclusions:**

Depending on the filter material and saturation, the likelihood of sevoflurane passing through passive flow carbon filters depends on the filter material and fresh gas flow. Combining the filter systems with anaesthetic gas scavenging systems could protect from pollution of ambient air with sevoflurane.

**Editor's key points**

• Passive flow anaesthetic gas capturing systems are potentially useful to reduce operating room and environmental pollution, but their performance is unclear.
• Sevoflurane binding capacity and resistive pressure were determined during simulated ventilation at different stages of filter saturation and fresh gas flow was tested for three carbon filter materials.
• Sevoflurane passage through passive flow carbon filters depended on the filter material and fresh gas flow.
• Sevoflurane concentrations in filtered gas measured at randomly selected clinical anaesthesia workstations was generally less than 10 ppm in unsaturated filters.
• Combining the filter systems with anaesthetic gas scavenging systems could reduce release and pollution from exhaled sevoflurane.

Sevoflurane has a wide range of clinical applications because of its relatively short-acting effects, controllability of concentration, and good airway tolerance. However, exhaust gas from the anaesthesia machine is usually discharged into the atmosphere via an anaesthetic gas scavenging system. With respect to the contribution of sevoflurane to the global carbon footprint of the healthcare sector^1^ and to global warming,^2^^,^^3^ passive filter systems with activated carbon have been developed to capture sevoflurane in the exhaust gas.^4^ Although this technology has a long history in anaesthesia,^5^ little is known about the efficacy of currently available carbon filters.

The aim of the study was to test three types of activated carbon filter materials intended for use with sevoflurane for their adsorption capacity and efficiency in waste gas removal. We hypothesised that efficiency of carbon filter systems depends on the filter material used. In addition, seven operating theatres were randomly selected on a representative day to determine sevoflurane concentrations in the filtered air.

## Methods

Three types of passive flow systems with activated carbon were tested: CONTRAfluran® (ZeoSys Medical GmbH, Luckenwalde, Germany), FlurAbsorb® (Sedana Medical AB, Danderyd, Sweden), and Anaesthetic Agent Filter 633 (AAF633, Drägerwerk AG & Co. KGaA, Lübeck, Germany). CONTRAfluran and FlurAbsorb come in rigid plastic containers of comparable dimensions with a filling quantity of ∼1000 g each. These containers are equipped with a DIN ISO 15 inflow connector and a perforated button plate to let the gas flow out. A thin foam layer at the top and the button inside the canister is supposed to provide homogenous distribution of the gas and to prevent loss of carbon granules. AAF633 comes in smaller containers of ∼200 g. To achieve comparable conditions for all tested materials, we filled 1000 g filter material of AAF633 into cleaned CONTRAfluran containers for our experiments.

### Saturating and filter capacity

**Fig 1:**
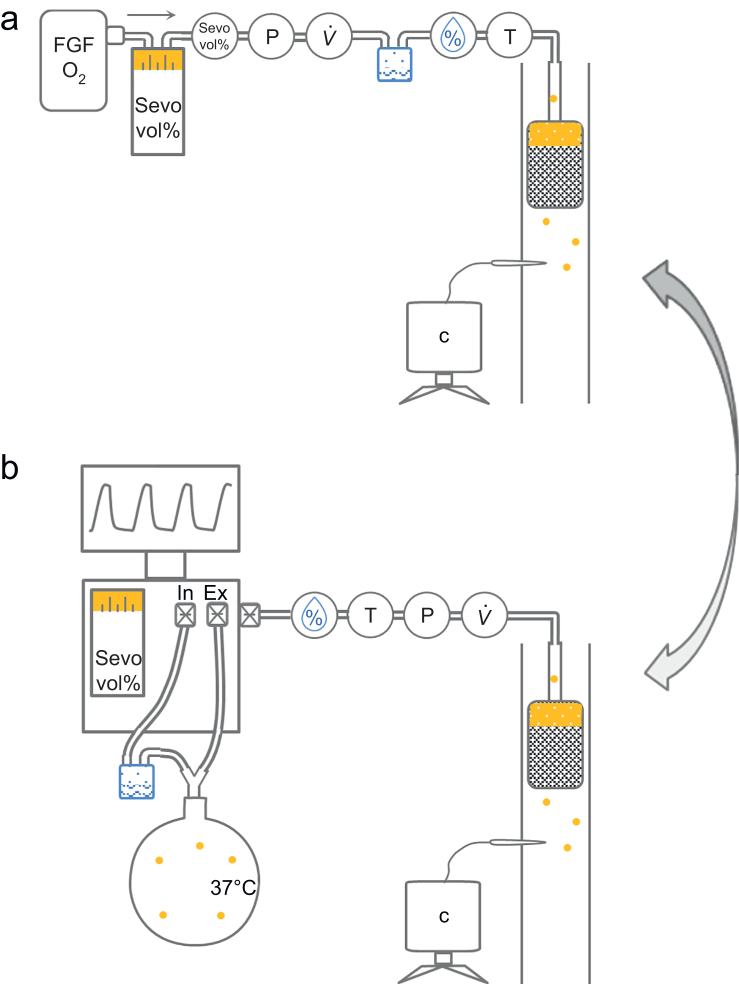
Schematic drawing of the physical model for saturation of filter materials (a) and breathing circuit to test filter efficiency under different clinical condition (b). c, concentration of sevoflurane; FGF, fresh gas flow; P, pressure; Sevo vol%, sevoflurane concentration; T, temperature; $\dot{V}$, flow; %, humidity.

Before starting the experiments, the weights of the filters were determined using an electronic precision balance (MSU6202S-1CE-DO, Sartorius Weighing Technology GmbH, Goettingen, Germany). A gas blender (Oxydig, Drägerwerk AG & Co. KGaA) was used to generate a gas flow that streamed through a sevoflurane vaporiser (Vapor 2000, Drägerwerk AG & Co. KGaA) and a humidifier (MR850, Fisher & Paykel Healthcare Limited, Auckland, New Zealand) to mimic physiological conditions of breathing gas before leading into the carbon filters (Fig. 1a). Flow, pressure, and sevoflurane concentration were measured between the vaporiser and the humidifier using FlowAnalyser (PF-301 VAC, IMT Analytics AG, Buchs, Switzerland) in combination with MultiGasAnalyser (OR-703, IMT Analytics AG). Temperature and flow were measured between the humidifier and filter (OHT20-A, Omni Elektronik GmbH, Lindlar, Germany). Additionally, concentration of sevoflurane was measured at the outlet of the respective carbon filter container using a highly sensitive gas analyser (GT5000 TERRA, Gasmet Technologies GmbH, Karlsruhe, Germany), which was calibrated according to the manufacturer's specifications. The GT5000 covers a measuring range for sevoflurane up to 50 ppm.

Carbon filters were partially saturated stepwise, each step followed the same protocol: fresh gas flow (FGF) was set to 10 L min^−1^ at a fraction of inspired oxygen of 0.4. The vaporiser was adjusted to achieve a concentration of sevoflurane 6 vol% in the mainstream measurement site. Sevoflurane-enriched gas was then heated and humidified by passing it through the humidifier to reach body temperature pressure saturated (BTPS) conditions (37^o^C, ambient pressure, air saturated with water vapour) before entering the filter. Each filter was streamed for 14 min at each saturation step. With respect to otherwise constant conditions, the volume of vaporised sevoflurane during a single saturation step was calculated as 10 L min^−1^×6 vol%×14 min=8400 ml. Under standard temperature and pressure, dry (STPD) conditions (i.e. dry air, 20°C room temperature, and atmospheric pressure), gaseous sevoflurane 184 ml translates into liquid sevoflurane 1 ml.^6^ Accordingly, the volume of liquid sevoflurane applied during a single saturation step was 8400 ml/184 ml=45.7 ml. After each saturation step the filter was weighed. The difference between the gain in filter weight during the saturation step and the amount of sevoflurane was calculated as the amount of water adsorbed by the filter material.

After each step, filter containers were connected to an anaesthesia machine in order to perform measurements under simulated ventilation of an anaesthetised patient (see below), and the next saturation step followed. Saturation steps were repeated until the concentration of sevoflurane at the outlet of the container exceeded 10 ppm, at which the filter was considered fully saturated. This limit was chosen as it represents the threshold value for sevoflurane concentration in the workplace.^7^, ^8^, ^9^

### Filter efficiency during simulated ventilation

We used an anaesthesia machine (Primus, Drägerwerk AG & Co. KGaA) and breathing tubes (DAR, Covidien LLC, Mansfield, MA, USA) to ventilate a physical model of an adult patient. To humidify the breathing gas, a humidifier (MR850, Fisher & Paykel) was inserted into the inspiratory limb of the breathing circuit and the inspiratory hose was heated to 37°C with an externally applied heating wire. The lung model consisted of a glass bottle with a volume of 25 L corresponding to a compliance of 25 ml cm H_2_O^−1^ (Fig. 1b).^10^

Carbon filter containers were connected to the waste gas outlet of the anaesthesia machine using a standard 22-mm plastic scavenging hose. Pressure (P), flow ($\dot{V;}$ SFM3300-AW, Sensirion AG, Stäfa, Switzerland), humidity, and temperature (OHT20-A) were measured at the end of the scavenging hose with the sensors directly connected to the inlet of the filter. Characteristic measures of the curves (e.g. peak pressure during coughing) were calculated using MatLab (R2022b, The MathWorks Inc., Natick, MA, USA).

In order to avoid interference of environmental air flow with our measurements, filter canisters were placed inside a circular acrylic glass cylinder (inner diameter 140 mm) open to atmosphere at the top and bottom. Concentration of sevoflurane in the air was measured 30 cm beneath the filter using the GT5000 gas analyser (Fig. 1b).

The model was ventilated in the volume-controlled mode with a tidal volume of 500 ml, breathing frequency of 12 bpm, FiO_2_ of 0.4 and positive end-expiratory pressure (PEEP) of 5 cm H_2_O. For intervals of 2 min each, FGF was set to 0.5 L min^−1^, 2 L min^−1^, and 10 L min^−1^ at an end-expiratory sevoflurane concentration of 2 vol%. At the end of each ventilation phase, coughing was simulated by rapid insufflation of 1 L of air into the breathing system. Thereafter, the concentration of released sevoflurane was measured behind the respective carbon filter at an FGF of 2 L min^−1^ and 10 L min^−1^ without sevoflurane in the breathing circuit. Between each ventilation setting, the acrylic glass cylinder was flushed with fresh air to rule out the possibility of residual sevoflurane influencing the measurement in the ambient air. All measurements were repeated three times after complete dismantling and rebuilding of the setup.

### Filter performance in the clinical setting

In order to assess filter performance in the clinical environment, the concentration of sevoflurane was measured in randomly selected anaesthesia induction rooms equipped with a Primus anaesthesia machine and passive CONTRAfluran filters. Ethical approval was requested for these measurements but deemed not necessary by the local Ethics Committee.

After induction of anaesthesia in a random patient, mechanical ventilation was maintained with FGF 2 L min^−1^, and sevoflurane concentration of 2 vol% according to clinical standards and independent from this study. To avoid any influence of air conditioning in the room on the measurements, the respective filter container was placed in an acrylic cylinder and sevoflurane concentration measurements were performed as described above.

The degree of saturation of the respectively present filter in clinical use was determined by its weight: the weight of the unused CONTRAfluran filters (m) showed low variability (m = 1037 [7] g) and according to our laboratory findings were considered fully saturated after uptake of sevoflurane 280 g. Consequently, degree of saturation was calculated from the weight of the filter (m_Filter_) according to Eq. (1):

(1) $$\text{Degree}\;\text{of}\;\text{saturation}\;\left(\%\right)=\frac{m_{Filter}-1037\;g}{280\;g}\;\times 100$$

### Statistical analysis

Data are presented as mean (sd) or boxplot indicating mean, upper and lower quartile, and minimal and maximal values, if not indicated otherwise. Values of sevoflurane concentration above 50 ppm are given as ‘>50 ppm’, because of the upper measurement limit of the GT5000. Differences between filters and settings were analysed using two-way analysis of variance (anova) followed by Tukey's multiple comparisons test, if appropriate. Differences between resistive pressure were analysed using one-way anova followed by Tukey's multiple comparisons test, if appropriate.

## Results

**Fig 2:**
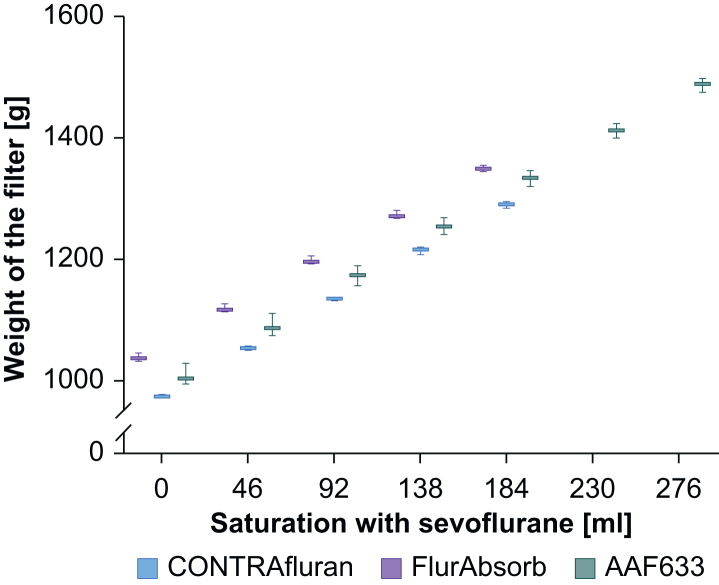
Filter weight depending on saturation of the filter with sevoflurane (BTPS conditions) for CONTRAfluran (ZeoSys Medical GmbH), FlurAbsorb (Sedana), and AAF633 (Dräger). Box plot indicates mean, upper and lower quartile, and minimal and maximal values (*n*=3). BTPS, body temperature pressure saturated.

CONTRAfluran filters weighed 1037 (7) g, FlurAbsorb filters weighed 973 (1) g, and filters 633 weighed 1007 (18) g. The weights of all filters increased almost linearly with each saturation step (Fig. 2). At BTPS conditions the amount of water adsorbed accounted for ∼8.6% of the total gain in weight, independent of the filter in question.

At 0.5 and 2 L min^−1^ FGF, sevoflurane concentration in the filtered exhaust gas was lowest with the AFF633 compared with the other filters at all saturation levels (all *P*<0.007; Table 1). At a FGF of 10 L min^−1^, sevoflurane concentration was >10 ppm at each saturation level with the FlurAbsorb filter, after four saturation levels (corresponding to 184 ml sevoflurane) with the CONTRAfluran filter and after six saturation levels (corresponding to 276 ml sevoflurane) with the AAF633 (Table 1). During simulated coughing, sevoflurane concentration in the filtered exhaust gas did not exceed 10 ppm in any filter (Table 1).

**Table 1:** Concentration of sevoflurane (c) and exhaust gas flow ($\dot{V}$) measured at different ventilation settings and saturation steps. Values are given as mean (SD). FGF, fresh gas flow.

Saturation	Sevoflurane (vol%)	FGF (L min^−1^)	CONTRAfluran®		FlurAbsorb®		AAF633	
			c (ppm)	$\dot{V}$ (L min^−1^)	c (ppm)	$\dot{V}$ (L min^−1^)	c (ppm)	$\dot{V}$ (L min^−1^)
0 ml (0 g)	2	0.5	2.8 (1.2)	−0 (0.1)	2.3 (0.5)	0 (0)	1.4 (0.3)	0 (0)
	2	2.0	2.6 (0.7)	−1.7 (0)	3.7 (0.8)	−1.9 (0)	1.0 (0.4)	−1.9 (0.1)
	2	10	3.5 (0.3)	−10.8 (0.1)	28.1 (9.1)	−10.9 (0.1)	6.2 (9.5)	−10.8 (0)
	2	Cough	3.8 (0.6)	−74.2 (6.8)	6.4 (0.9)	−65.3 (12)	2.4 (0.4)	−84.0 (11.2)
	0	2.0	1.6 (0.2)	−1.6 (0.1)	2.1 (0.2)	−1.8 (0)	2.1 (1.4)	−1.7 (0.1)
	0	10	1.3 (0.3)	−9.5 (0.1)	1.9 (0.2)	−9.6 (0)	1.6 (0.5)	−9.4 (0)
46 ml (70 g)	2	0.5	2.7 (0.3)	0 (0)	2.5 (0.3)	0 (0)	1.2±0.2	−0.1 (0.1)
	2	2.0	2.6 (0.4)	−1.3 (1.1)	3.8 (0.3)	−1.4 (0.1)	1.1±0.2	−1.9 (0)
	2	10	2.2 (1.4)	−10.8 (0)	19.2 (4.1)	−11.9 (0.1)	9.7 (14.8)	−10.7 (0)
	2	Cough	3.9 (0.4)	−82.3 (29.3)	6.2 (1.8)	−63.9 (9.2)	2.4 (1.0)	−85.6 (12.4)
	0	2.0	1.8 (0.3)	−1.5 (0)	2.5 (0.4)	−1.8 (0)	1.3 (0.4)	−1.5 (0)
	0	10	1.2 (0.2)	−9.1 (0.6)	1.9 (0.7)	−9.6 (0.1)	2.6 (1.9)	−9.4 (0)
92 ml (140 g)	2	0.5	2.5 (0.3)	0 (0)	2.7 (0.3)	0 (0)	1.2 (0.3)	−0.1 (0.3)
	2	2.0	2.5 (0.2)	−1.7 (0.1)	4.8 (0.4)	−2.0 (0.1)	1.1 (0.4)	−1.8 (0.2)
	2	10	3.2 (0.4)	−10.8 (0.1)	23.8 (4.1)	−10.9 (0.1)	10.6 (14.3)	−10.8 (0.3)
	2	Cough	4.0 (0.2)	−76.5 (22.2)	6.1 (0.5)	−76.7 (4.5)	2.7 (0.9)	−69.0 (10.1)
	0	2.0	1.8 (0.2)	−1.6 (0)	2.6 (0.4)	−1.8 (0)	1.4 (0.3)	−1.6 (0.1)
	0	10	1.5 (0.3)	−9.3 (0.5)	1.9 (0.2)	−9.7 (0.1)	0.9 (0.2)	−9.3 (0.1)
138 ml (210 g)	2	0.5	2.3 (0.6)	0 (0)	2.6 (0.6)	0 (0)	1.0 (0.3)	−0.1 (0.1)
	2	2.0	2.2 (0.3)	−1.6 (0.2)	4.0 (1.5)	−1.9 (0.1)	1.1 (0.3)	−1.8 (0.2)
	2	10	2.5 (0.3)	−10.7 (0.1)	23.8 (7.9)	−10.8 (0.3)	1.5 (0.7)	−10.7 (0.2)
	2	Cough	3.9 (1.5)	−57.4 (13.4)	7.8 (1.4)	−77.7 (12.5)	2.3 (0.8)	−77.4 (3.4)
	0	2.0	1.4 (0.7)	−1.5 (0.1)	2.4 (0.6)	−1.8 (0)	1.2 (0.4)	−1.6 (0.2)
	0	10	1.1 (0.8)	−9.6 (0)	1.8 (0.2)	−9.6 (0)	0.8 (0.3)	−9.5 (0.1)
184 ml (280 g)	2	0.5	2.4 (0.6)	0 (0)	2.6 (0.4)	0 (0)	1.3 (0.3)	−0.1 (0.1)
	2	2.0	2.5 (0.6)	−1.8 (0)	3.7 (0.6)	−1.9 (0.1)	1.3 (0.4)	−1.8 (0.2)
	2	10	27.0 (10.8)	−10.7 (0.1)	22.4 (3.4)	−10.9 (0)	8.7 (12.4)	−10.8 (0.1)
	2	Cough	4.8 (0.3)	−99.0 (32.3)	6.4 (1.2)	−86.0 (10.9)	2.2 (0.9)	−88.6 (11.3)
	0	2.0	2.3 (0.4)	−1.6 (0)	2.3 (0.9)	−2.1 (0.3)	1.5 (0.1)	−1.6 (0.2)
	0	10	24.7 (4.3)	−9.5 (0.1)	2.2 (0.5)	−9.6 (0)	2.2 (2.3)	−9.4 (0)
230 ml (350 g)	2	0.5					1.5 (0.1)	−0.1 (0.1)
	2	2.0					1.3 (0.4)	−1.8 (0.2)
	2	10					5.4 (7.6)	−10.7 (0)
	2	Cough					2.7 (1.4)	−75.0 (11.3)
	0	2.0					1.3 (0.1)	−1.6 (0.2)
	0	10					1.1 (0.1)	−9.4 (0.1)
276 ml (420 g)	2	0.5					1.7 (0.7)	−0.1 (0.1)
	2	2.0					1.3 (0.7)	−1.7 (0.2)
	2	10					9.9 (7.5)	−10.5 (0.3)
	2	Cough					3.4 (1.1)	−84.2 (13.3)
	0	2.0					1.5 (0.3)	−1.6 (0.1)
	0	10					17.1 (17.7)	−6.3 (5.4)

Without sevoflurane in the breathing circuit, sevoflurane concentration in the filtered exhaust gas was comparable to that during ventilation with sevoflurane at 2 L min^−1^ and 10 L min^−1^. Sevoflurane concentration exceeded 10 ppm at FGF 10 L min^−1^ without sevoflurane after four saturation levels with the CONTRAfluran filter and six saturation levels with the AAF633 filter.

**Fig 3:**
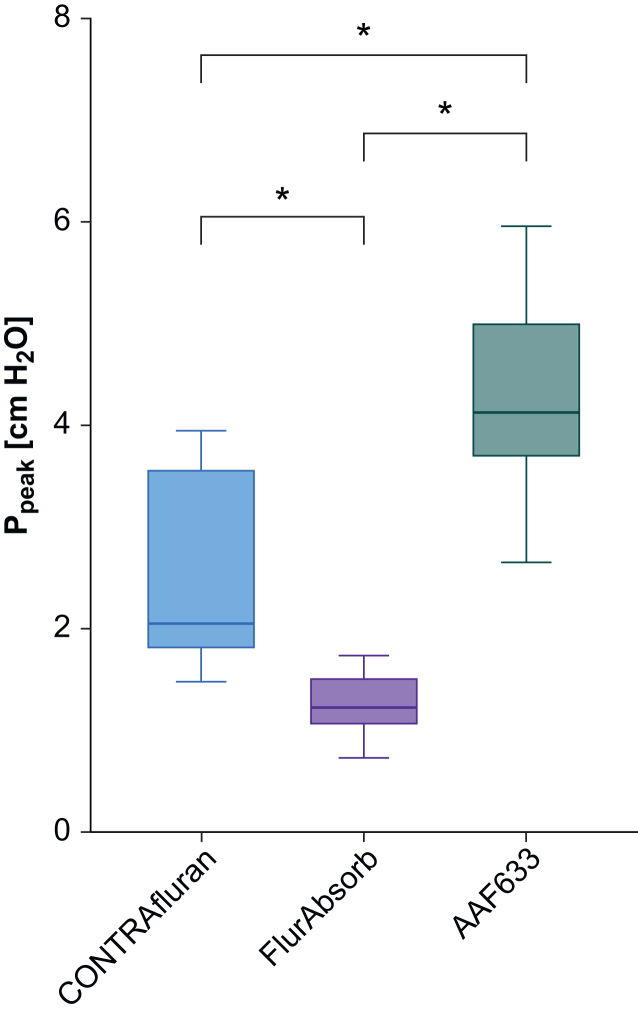
Peak pressure (P_peak_) during simulated coughing for CONTRAfluran, FlurAbsorb, and AAF633. Box plot indicates mean, upper and lower quartile, and minimal and maximal values (*n*=3). One-way analysis of variance followed by Tukey's multiple comparison test. ∗*P*<0.001.

During simulated ventilation, exhaust gas entering the filter at the end of the scavenging hose had a temperature of 26 (1)°C and a humidity of 61 (2)% on average. The resistance pressure decrease across the filters did not exceed 0.2 (0.1) cm H_2_O during ventilation, but increased up to 5.2 (0.2) cm H_2_O during simulated coughing (Fig. 3, Supplementary Table S1). The resistance pressure decrease differed significantly between all filters (all *P*<0.001), and was lowest for FlurAbsorb and highest for AAF633.

### Filter performance in the clinical setting

In random measurements in routine clinical practice, the weight of four of the seven CONTRAfluran filters exceeded the value considered fully saturated in our laboratory study (Table 2). In two of the seven cases, sevoflurane concentration in filtered exhaust gas exceeded 10 ppm.

**Table 2:** Concentrations of sevoflurane (c) during clinical application of CONTRAfluran filters were calculated depending on degree of saturation of the filters saturated in the physical model. Ventilation settings: fresh gas flow=2 L min^−1^, sevoflurane 2 vol%, tidal volume=500 ml, frequency=12 min^−1^, FiO_2_=0.4, and positive end-expiratory pressure=5 cm H_2_O. Measurement of patient 3 was performed with fresh gas flow=3 L min^−1^ because of patient safety concerns.

Patient No.	Weight of the filter (g)	Degree of saturation (%)	c (ppm)
1	1360	105	0.1
2	1328	95	1.2
3	1435	129	35.6
4	1457	136	1.6
5	1491	147	>50
6	1059	7	1.9
7	1137	32	1.6

## Discussion

Our main findings can be summarised as: (1) activated anaesthesia carbon gas filters can keep ambient air sevoflurane concentrations below the specified occupational exposure limits if the individual maximum filter capacity and low fresh gas flows are maintained; (2) relevant quantities of sevoflurane pass through the filter at high FGF rates; (3) the binding capacity depends on the filter material used and can be considerably lower than specified in the manufacturers' instructions; (4) during ventilation without anaesthetic gas, small amounts of sevoflurane are released from the filters; and (5) at high peak flow, the filters can offer considerable resistance to air flow, which depends on the nature of the filter material, but not on the state of saturation.

The main purpose of activated carbon filters is to adsorb anaesthetic gases such as sevoflurane from the anaesthetic gas delivery system during general anaesthesia. This is necessary not only to avoid environmental damage by preventing sevoflurane from escaping into the atmosphere, but also to prevent accumulation of relevant concentrations of sevoflurane in the ambient air and to protect operating room staff from potential health hazards.

Passive flow activated carbon anaesthetic gas filters can keep the concentration of sevoflurane in the ambient air below their accepted occupational exposure limits, provided that the filters are used below their maximum binding capacity. The binding capacities for commercially available filter systems (Sedaconda, ZeoSys Medical GmbH) are specified by the manufacturers. However, our results show considerable differences between the observed sevoflurane uptake capacity and the manufacturer's specifications. For CONTRAfluran, an uptake capacity of ∼400 g sevoflurane is stated by the manufacturer (which corresponds to ∼260 ml given the specific weight of sevoflurane of 1.53 g ml^−1^.^11^ In our experiments, the critical release was already reached at ∼76% of this value. For FlurAbsorb, the manufacturer states a binding capacity of sevoflurane 500 ml,^12^ but at our threshold value not even half of the stated capacity was reached.

The differences in binding capacities might be because of different thresholds used by the manufacturers and in our environment. We are not aware of the manufacturers' standards, but we used 10 ppm as the critical limit for sevoflurane concentration in ambient air. This limit is supported by national recommendations for occupational workplace safety in Austria,^13^ Poland, Finland, Sweden, and Norway.^7^ Although not all countries specify limits for sevoflurane, clinicians believe that the ambient air concentration of anaesthetic gases should be as low as possible. Regarding the approach to measure ambient air concentrations of sevoflurane, we excluded the potential dilution effects of air conditioning, as we believe that air conditioning is not available in all anaesthesia workplaces. Passive flow activated carbon filters are of particular interest for workplaces where no flushing systems are available (i.e. workplaces that might not meet operating room standards, including lack of air conditioning).

The humidity of the breathing air could be another reason for the observed differences in binding capacity. We passed saturated air through the filters to simulate clinical conditions in a steady state and found 8.6% of the filters' weight gain was accounted for by water.

During simulated ventilation, humidity and temperature of the exhausted gas had dropped in comparison to the saturated conditions in the lung model. This could be a result of the relatively long distance the expiratory breathing gas travelled through the breathing hose, the machine, and the scavenging hose. To the best of our knowledge, no objective data on the humidity and temperature of waste gas are available. The lower humidity observed in our simulated clinical setting increased the filter's binding capacity for sevoflurane as acknowledged by some manufacturers.^14^

The binding capacity for sevoflurane might also vary with the ratio of time during which there is flow (during ventilation) or not (during device standby). Small amounts of sevoflurane were released from the partially saturated filters even though no anaesthetic gas was flowing. Accordingly, filter binding capacity for sevoflurane could appear higher if saturation occurs at periodic intervals. In the long term, this could give the impression that the total binding capacity is higher than found in our study. However, our aim was to demonstrate gas removal under the most demanding conditions, which we considered to be continuous flow through the filters under clinically representative conditions.

In contrast to our results, a recent study^15^ determined an overall removal efficiency for sevoflurane of 94.8% for an activated carbon filter system certified for clinical use in the UK. The investigators determined filter binding capacity and saturation by comparing the weights of filter cans and vaporisers at different stages. The high filter capacities determined in their study might be attributable to the fact that they did not consider a threshold value for release of sevoflurane into ambient air. In addition, use of dry air would have increased filter capacity for sevoflurane in their configuration.

**Fig 4:**
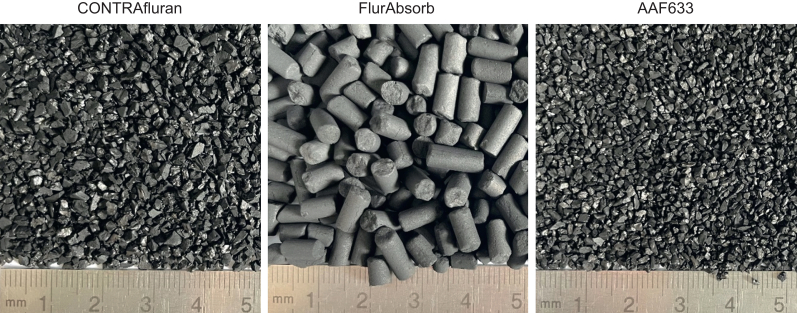
Photographs of filter materials. Filter material of CONTRAfluran is small granular. Filter material of FlurAbsorb is in the form of pellets. Filter material of AAF633 is smaller granules compared with CONTRAfluran.

The reasons for the overall difference in binding capacity between filters from different manufacturers are complex. As all materials were tested in filter cans of comparable size and internal structure, it can be assumed that the differences resulted from the filter material itself. The geometry of the carbon granule and the chemical properties of the activated carbon have an influence. A macroscopic examination of the granulate reveals relevant differences between the materials (Fig. 4). Taking into account granule size, the total surface area for adsorption is greatest for the Dräger AAF633 and least for the FlurAbsorb. In general, this corresponds to our results for the binding capacities of the filters, although the differences were less clear than they might appear when viewed macroscopically. With regard to granule size, the density of the granule arrangement in the filter can have an influence on the distribution of flow within the filter. We found that the FlurAbsorb passes sevoflurane at high FGF but low filter saturation. Considering the comparatively large intergranular space in this material, the exhaust gas can pass easily and with short contact time. This assumption is supported by the overall lowest airflow resistance of the filters.

The resistance to airflow was low for all filters at continuous flow, regardless of the stage of filter saturation. However, it increased up to a resistive pressure gradient of 5 cm H_2_O with the AAF633, the material of the finest granule size. Although such a transient increase in pressure might have only a small effect on airway pressure load during coughing, obstruction of the scavenging outlet could result in serious technical malfunctions of the anaesthesia workstation.^16^^,^^17^

Sevoflurane concentrations remained within our limits at higher flow rate during simulated coughing. Coughing produces a high peak flow rate, but the absolute displaced volume of the cough is limited. It could be speculated that the amount of displaced volume remained in the filter long enough to be cleared of sevoflurane at an FGF of 2 L min^−1^ in advance. In this context, the low clearance rate for sevoflurane with the FlurAbsorb filter needs further examination. FlurAbsorb filters are intended for use during inhaled sedation with an open circuit ventilator and sevoflurane in the ICU. Therefore, the typical flow rate passing the filter is in the range of minute ventilation under such conditions.

CONTRAfluran is still the only filter system approved for perioperative use in Germany. For clinical use, the canister is placed in a holder that contains an optical fill level indicator (SENSOflurane, ZeoSys Medical Gmbh). In our experiments, we intentionally saturated the filters above our threshold, and in the clinical measurements four out of seven filters were already saturated above the threshold. However, neither during our laboratory experiments nor during the clinical measurements did the system's fill sensor indicate complete saturation of the filters. This was not an endpoint of our study and we are not aware of the saturation limits defined by the manufacturer.

### Limitations of the study

The transfer of our results to the clinical environment should take the influence of carbon dioxide into account. We deliberately did not add carbon dioxide to sevoflurane and moisture, as with a third variable, differentiation of the substances contributing to weight gain of the filters would not have been possible. In additional experiments (not shown) we streamed the filters with dry air and physiological end-expiratory concentrations of carbon dioxide and found a maximum of 0.5% in weight gain, such that including CO_2_ would not likely have influenced our results to a relevant extent.

We considered 10 ppm of sevoflurane in the filtered air, a critical threshold for filter capacity. Measurements were performed, however, close to the filter and do not necessarily mirror the concentration of sevoflurane relevant for occupational safety. A study evaluating air pollution with sevoflurane found far lower concentrations in the breathing zone of ICU personnel.^18^ Peak sevoflurane concentrations in the ambient air depend on numerous factors such as room size, presence of air conditioning system, and disconnection of the breathing circuit, for example.^18^^,^^19^ Because of a lack of standardisation for these factors, we considered sevoflurane measurements close to the filter the most reliable approach to compare the performance of different filter systems.

Our findings also raise new questions. While we have evaluated the filters during constant flow and sevoflurane concentrations typical during anaesthesia, decelerating flow and lower concentrations of sevoflurane as expected during inhalation sedation in the ICU could show different filter performance. With the observed dependency on flow, dimensions of the filter canisters or granule size might be optimised for the respective application. Moreover, it can be hypothesised that the saturated filters not only release sevoflurane when streamed with air, but also when exposed to atmosphere or during transport.

In conclusion, our results indicate that activated carbon filters sufficiently adsorb sevoflurane depending on the filter material and fresh gas flow. Maximum binding capacity was influenced by moisture and lower than expected from manufacturers' specifications. Therefore, we recommended monitoring filter saturation electronically or combining the passive flow filters with an anaesthetic gas scavenging system. Further studies are needed to assess workplace safety with carbon filter systems.

## Authors’ contributions

Conception and design of the study: CW, BF, TL, SS, JS

Acquisition of data: CW, BF, JS

Analysis and interpretation of data: CW, BF, TL, SS, JS

Drafting and revision the manuscript: CW, BF, TL, SS, JS

Approved the final version of the manuscript: all authors

## Acknowledgements

The authors would like to thank, F. Ulbrich and C. Lehane for supporting the study and K. Klein for technical support during the study.

## Declaration of interest

The authors declared that they have no conflicts of interest.

## References

[1] Richter H., Weixler S., Schuster M. (2020). The carbon footprint of anaesthesia. How the choice of volatile anaesthetic affects the CO_2_ emissions of a department of anaesthesiology. Anästh Intensivmed.

[2] Ryan S.M., Nielsen C.J. (2010). Global warming potential of inhaled anesthetics: application to clinical use. Anesth Analg.

[3] Vollmer M.K., Rhee T.S., Rigby M. (2015). Modern inhalation anesthetics: potent greenhouse gases in the global atmosphere. Geophys Res Lett.

[4] Hinterberg J., Beffart T., Gabriel A. (2022). Efficiency of inhaled anaesthetic recapture in clinical practice. Br J Anaesth.

[5] Kim B.M., Sircar S. (1977). Adsorption characteristics of volatile anesthetics on activated carbons and performance of carbon canisters. Anesthesiology.

[6] Biro P. (2014). Calculation of volatile anaesthetics consumption from agent concentration and fresh gas flow. Acta Anaesthesiol Scand.

[7] GESTIS Datenbank der DGUV IGfcS. https://ilv.ifa.dguv.de/limitvalues/54107.

[8] Deutsche Gesetzliche Unfallversicherung (DGUV) (2021).

[9] Gerding J., Eickmann U. (2019).

[10] Wenzel C., Frey C., Schmidt J., Lozano-Zahonero S., Urban G., Schumann S. (2020). A linearized expiration flow homogenizes the compartmental pressure distribution in a physical model of the inhomogeneous respiratory system. Physiol Meas.

[11] ZeoSys Medical GmbH (2022). https://zeosys-medical.de/wp-content/uploads/2021/08/CONTRAfluran-Instruction-for-Use_EN_082021.pdf.

[12] Sedanamedical Ltd (2024). https://sedanamedical.com/de/produkte/flurabsorb/flurabsorb-s/.

[13] (2016). https://auva.at/praevention/medien-und-publikationen/publikationen/m-135-sicherer-umgang-mit-narkosegasen.

[14] Sedana Medical Ltd (2024). https://sedanamedical.com/de/produkte/sedaconda-acd/sedaconda-acd-s.

[15] Vaghela M., Kay R.H., Jones L., Greig P. (2023). Inhalational anaesthetics: an assessment of agent delivery and capture. Anaesthesia.

[16] Elakkumanan L.B., Vasudevan A., Krishnappa S., Pandey R.R., Balachander H., Badhe A.S. (2012). Obstruction to scavenging system tubing. J Anaesthesiol Clin Pharmacol.

[17] Vinayagam S., Dhanger S., Thomas D., Venkatesh Babu T.A. (2018). Scavenging tubing compression: a rare cause for anaesthesia ventilator malfunction. Indian J Anaesth.

[18] Herzog-Niescery J., Vogelsang H., Gude P. (2019). Environmental safety: air pollution while using MIRUS™ for short-term sedation in the ICU. Acta Anaesthesiol Scand.

[19] Gandhi J., Baxter I. (2023). Efficiency of inhaled anaesthetic recapture in clinical practice. Comment on Br J Anaesth 2022; 129: e79-81. Br J Anaesth.

