# Supplementary material for: Efficiency of passive activated carbon anaesthetic gas capturing systems during simulated ventilation
Source: Br J Anaesth. 2024 Jul 2;133(6):1518–24. doi: 10.1016/j.bja.2024.05.028 (PMC11589552; doi:10.1016/j.bja.2024.05.028)
Supplement: Multimedia component 1 [file mmc1.docx]

**Supplementary Table ST1** Measured resistive back pressure (P) measured for different ventilation settings and saturation steps. Values are given as mean±SD. FGF = Fresh gas flow.

|  |  |  |  | CONTRAfluran |  | FlurAbsorb |  | AAF633 |
| --- | --- | --- | --- | --- | --- | --- | --- | --- |
| Saturation | Sevoflurane  [Vol.-%] | FGF  [l min^-1^] |  | P  [cmH_2_O] |  | P  [cmH_2_O] |  | P  [cmH_2_O] |
| 0 ml (0 g) | 2 | 0.5 |  | 0±0 |  | 0±0 |  | 0±0 |
|  | 2 | 2.0 |  | 0±0 |  | 0±0 |  | 0±0 |
|  | 2 | 10 |  | 0±0 |  | 0±0 |  | 0.2±0 |
|  | 2 | Cough |  | 2.0±0 |  | 0.9±0.2 |  | 4.6±1.0 |
|  | 0 | 2.0 |  | 0±0 |  | 0±0 |  | 0±0 |
|  | 0 | 10 |  | 0±0 |  | 0±0 |  | 0.2±0 |
|  |  |  |  |  |  |  |  |  |
| 46 ml (70 g) | 2 | 0.5 |  | 0±0 |  | 0±0 |  | 0±0 |
|  | 2 | 2.0 |  | 0±0 |  | 0±0 |  | 0±0 |
|  | 2 | 10 |  | 0±0 |  | 0±0 |  | 0.2±0 |
|  | 2 | Cough |  | 2.7±2.7 |  | 1.5±0.3 |  | 4.2±0.8 |
|  | 0 | 2.0 |  | 0±0 |  | 0±0 |  | 0.1±0.1 |
|  | 0 | 10 |  | 0±0 |  | 0±0 |  | 0.2±0 |
|  |  |  |  |  |  |  |  |  |
| 92 ml (140 g) | 2 | 0.5 |  | 0±0 |  | 0±0 |  | 0±0 |
|  | 2 | 2.0 |  | 0±0 |  | 0±0 |  | 0.1±0.1 |
|  | 2 | 10 |  | 0±0 |  | 0±0 |  | 0.3±0 |
|  | 2 | Cough |  | 2.6±0.9 |  | 1.2±0.2 |  | 3.3±0.7 |
|  | 0 | 2.0 |  | 0±0 |  | 0±0 |  | 0±0 |
|  | 0 | 10 |  | 0±0 |  | 0±0 |  | 0.2±0 |
|  |  |  |  |  |  |  |  |  |
| 138 ml (210 g) | 2 | 0.5 |  | 0±0 |  | 0±0 |  | 0±0 |
|  | 2 | 2.0 |  | 0±0 |  | 0±0 |  | 0±0 |
|  | 2 | 10 |  | 0±0 |  | 0±0 |  | 0.2±0 |
|  | 2 | Cough |  | 1.8±0.5 |  | 1.3±0.2 |  | 4.3±0.6 |
|  | 0 | 2.0 |  | 0±0 |  | 0±0 |  | 0±0 |
|  | 0 | 10 |  | 0±0 |  | 0±0 |  | 0.2±0 |
|  |  |  |  |  |  |  |  |  |
| 184 ml (280 g) | 2 | 0.5 |  | 0±0 |  | 0±0 |  | 0±0 |
|  | 2 | 2.0 |  | 0±0 |  | 0±0 |  | 0±0 |
|  | 2 | 10 |  | 0.1±0 |  | 0±0 |  | 0.2±0.1 |
|  | 2 | Cough |  | 2.9±1.3 |  | 1.5±0.2 |  | 5.2±0.2 |
|  | 0 | 2.0 |  | 0±0 |  | 0±0 |  | 0±0 |
|  | 0 | 10 |  | 0.1±0 |  |  |  | 0.2±0.1 |
|  |  |  |  |  |  |  |  |  |
| 230 ml (350 g) | 2 | 0.5 |  |  |  |  |  | 0±0 |
|  | 2 | 2.0 |  |  |  |  |  | 0±0 |
|  | 2 | 10 |  |  |  |  |  | 0±0 |
|  | 2 | Cough |  |  |  |  |  | 4.1±1.7 |
|  | 0 | 2.0 |  |  |  |  |  | 0±0 |
|  | 0 | 10 |  |  |  |  |  | 0.2±0 |
|  |  |  |  |  |  |  |  |  |
| 276 ml (420 g) | 2 | 0.5 |  |  |  |  |  | 0±0 |
|  | 2 | 2.0 |  |  |  |  |  | 0±0 |
|  | 2 | 10 |  |  |  |  |  | 0.2±0 |
|  | 2 | Cough |  |  |  |  |  | 4.1±0.4 |
|  | 0 | 2.0 |  |  |  |  |  | 0±0 |
|  | 0 | 10 |  |  |  |  |  | 0.2±0 |
